# Letter to the Editor: “Ion Channels in Brain Metastasis”—Ion Channels in Cancer Set up and Metastatic Progression

**DOI:** 10.3390/ijms18040718

**Published:** 2017-03-28

**Authors:** Daniela D’Arcangelo, Ezio M. Nicodemi, Antonio Facchiano

**Affiliations:** Istituto Dermopatico dell’Immacolata, Istituto Dermopatico dell’Immacolata, Istituto di Ricovero e Cura a Carattere Scientifico (IDI-IRCCS), Fondazione Luigi Maria Monti (FLMM), via Monti di Creta 104, 00167 Rome, Italy; d.darcangelo@idi.it (D.D.A.); e.nicodemi@idi.it (E.M.N.)

## To the Editor,

The review by Klumpp, L. et al. entitled Ion Channels in Brain Metastasis [1] discusses the role of ion channels in breast cancer, lung cancer and melanoma in metastatic tropism to the brain. The Authors debate the important role of ion channels in different steps of metastasis to the brain. Ion channels have shown aberrant expression in cancer cells and regulate tumor transformation as well as progression or resistance to therapy. Currently, there is a large growing interest toward such a field of investigation. In fact, since the date of publication of Klumpp, L. et al.’s manuscript (from September 2016 to date—beginning of March 2017) 37 additional manuscripts appeared in PubMed having “ion channel” and “cancer” in the “Title” or “Abstract” fields. In such studies, a few other channels have been shown to be involved in cancer set up and progression, although not only focused on brain metastases from breast, lung and melanoma cancers. For instance, we [2] evaluated the expression of ninety ion-channel genes in 3673 human biopsies, in five different solid tumors (bladder cancer, breast cancer, glioblastoma, lung cancer and melanoma). We [2] and others [3,4,5,6,7,8,9] have shown a key role of ion channels in tumors common to Klumpp, L. et al., namely lung cancer, breast cancer and melanoma, as well as in other cancers such as glioblastoma, bladder cancer and colorectal cancer. These channels are calcium channel voltage-dependent (CACNA1D); FXYD domain-containing ion transport regulators (FXYD3, FXYD5); chloride intracellular channels (CLIC1); glutamate receptors (HTR3A); potassium channel voltage-gated channels (KCNE3, KCNE4, KCNN4); transient receptor potential cation channels (TRPA1, TRPC5, TRPM3, TRPV4) and Aquaporins (AQPs). Such a list may somehow integrate the scenario depicted by Klumpp, L. et al. As we underlined in our study, all such cancers have different histological origin but they have endothelial and vascular alterations in common. The role of ion channels in vascular alteration occurring in the metastatic process is clearly recognized but still not completely elucidated, as discussed by Klumpp, L. et al. Therefore, we believe that all these ion channels reported by Klumpp, L. et al. [1]., us [2] and others [3,4,5,6,7,8,9] may have a mechanistic role in the primary tumor set up as well as in the metastatic progression toward the brain and other organs.
